# The Role of miRNAs, miRNA Clusters, and isomiRs in Development of Cancer Stem Cell Populations in Colorectal Cancer

**DOI:** 10.3390/ijms22031424

**Published:** 2021-01-31

**Authors:** Victoria A. Stark, Caroline O. B. Facey, Vignesh Viswanathan, Bruce M. Boman

**Affiliations:** 1 Department of Biological Sciences, University of Delaware, Newark, DE 19716, USA; vstark@udel.edu (V.A.S.); vigneshv@stanford.edu (V.V.); 2 Center for Translational Cancer Research, Helen F. Graham Cancer Center & Research Institute, 4701 Ogletown-Stanton Road, Newark, DE 19713, USA; Caroline.Facey@christianacare.org; 3 Department of Radiation Oncology, Stanford University School of Medicine, Stanford, CA 94305, USA; 4 Department of Pharmacology & Experimental Therapeutics, Thomas Jefferson University, Philadelphia, PA 19107, USA

**Keywords:** microRNAs (miRNAs), colorectal cancer (CRC), cancer stem cell genes (CSCs), biomarker(s), *miRNA-17-92a* cluster, *miRNA-23b-27b-24* cluster, tumor heterogeneity

## Abstract

MicroRNAs (miRNAs or miRs) have a critical role in regulating stem cells (SCs) during development and altered expression can cause developmental defects and/or disease. Indeed, aberrant miRNA expression leads to wide-spread transcriptional dysregulation which has been linked to many cancers. Mounting evidence also indicates a role for miRNAs in the development of the cancer SC (CSC) phenotype. Our goal herein is to provide a review of: (i) current research on miRNAs and their targets in colorectal cancer (CRC), and (ii) miRNAs that are differentially expressed in colon CSCs. MicroRNAs can work in clusters or alone when targeting different SC genes to influence CSC phenotype. Accordingly, we discuss the specific miRNA cluster classifications and isomiRs that are predicted to target the *ALDH1*, *CD166*, *BMI1*, *LRIG1*, and *LGR5* SC genes. *miR-23b* and *miR-92A* are of particular interest because our previously reported studies on miRNA expression in isolated normal versus malignant human colonic SCs showed that *miR-23b* and *miR-92a* are regulators of the *LGR5* and *LRIG1* SC genes, respectively. We also identify additional miRNAs whose expression inversely correlated with mRNA levels of their target genes and associated with CRC patient survival. Altogether, our deliberation on miRNAs, their clusters, and isomiRs in regulation of SC genes could provide insight into how dysregulation of miRNAs leads to the emergence of different CSC populations and SC overpopulation in CRC.

## 1. Introduction

Our goal herein is to review current research findings on miRNAs in colorectal cancer (CRC), and to provide an update on miRNAs that target stem cell (SC) genes in CRC. We will first provide a brief discussion of the discovery of miRNAs and review of the canonical and noncanonical miRNA biogenesis pathways. Then, we will identify how dysregulation of miRNA biogenesis and/or function contributes to cancer. We then discuss and identify miRNAs and miRNA clusters that target SC genes or the genes involved in regulating SC properties. Finally, we provide bioinformatics information on miRNAs that are predicted to target SC genes in CRC, including their miRNA cluster classification and their isomiRs. The overall focus of this paper is to understand how dysregulation of miRNAs leads to emergence of cancer SC (CSC) populations and SC overpopulation in CRC.

## 2. Discovery of miRNAs

MicroRNAs are the class of short noncoding RNAs, approximately 18–25 nucleotides in length, which were identified based on their ability to regulate post-transcriptional gene expression. They are highly conserved across animal species, and essential for normal animal development and cellular processes such as proliferation, differentiation, viability and apoptosis [1,2]. Since their discovery, the role of miRNAs in pathogenesis including cancer, neurological disease, heart disease, autoimmune disease, viral disease, and bone disease has been well documented [3,4,5,6,7,8]. 

The first two miRNAs discovered were heterochronic genes essential for the timing of developmental events in *Caenorhabditis elegans* (*C. elegans*). *Lin-4* was the first miRNA to be identified by the Ambros and Ruvkun research teams in 1993 [9,10]. They found that *lin-4* had an essential role in the growth and differentiation of *C. elegans* hypodermic stem cells. *Lin-4* was later found to suppress gene expression of *lin-28* and *lin-14* by binding to the complementary regions of their 3′ UTRs [11,12]. Although these findings were quite exciting, this phenomenon seemed restricted to nematode development as there were no homologs found in vertebrates until a few years later with the discovery of a second miRNA, *let-7* [13,14]. While *lin-4* is essential for the transition of larvae from the first to second stages, *let-7* was found to be critical for the timing of later events from larval to the adult stage. However, unlike *Lin-4*, *Let-7* is highly conserved across animal species [13,14]. Overall, the significance and role of miRNAs in development and adult homeostasis have revolutionized the field of molecular biology and our understanding of pathogenesis of cancer and other diseases [12]. 

## 3. miRNA Biogenesis

It is important to understand miRNA biogenesis in order to elucidate how miRNA dysregulation causes cancer and promotes its progression. The biogenesis of miRNAs may involve a dominant canonical pathway or one of several noncanonical pathways [15].

### 3.1. Canonical Pathway 

**Figure 1:**
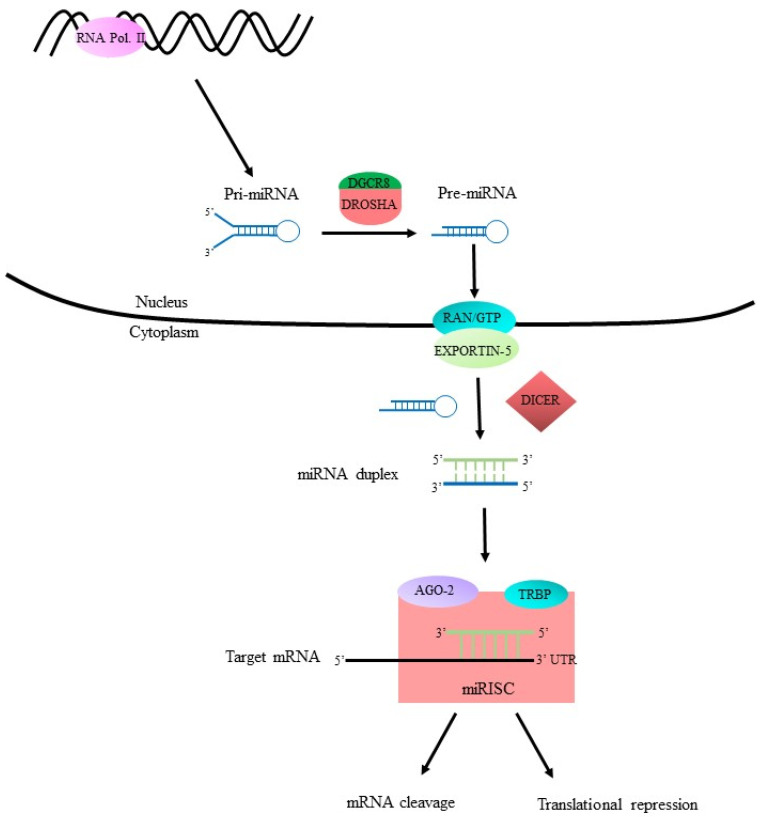
Canonical miRNA biogenesis pathway. RNA polymerase II transcribes DNA of interest into pri-miRNA containing ~80 nucleotides (nts). The Drosha/DGCR8 microprocessing complex cleaves pri-miRNA into pre-miRNA which contains ~65 nts. The Ran-GTP/Exportin-5 complex binds to pre-miRNA and exports it from the nucleus into the cytoplasm. Dicer aids in unwinding and transforming pre-miRNA into an miRNA duplex. The miRNA duplex is then further processed by RISC and Ago2 into mature miRNA containing ~22 nts. Then the miRNA-induced silencing complex miRISC either causes cleavage and degradation or translational repression of its target mRNA.

In the canonical pathway (Figure 1), biogenesis begins when primary transcript miRNA (pri-miRNA) is transcribed and then cleaved into precursor-miRNA (pre-miRNA) by a “microprocessor” complex consisting of the ribonuclease III (RNase III) enzyme Drosha and RNA binding protein DGCR8 [16,17,18,19]. The pre-miRNAs are then exported from the nucleus into the cytoplasm via the Ran-GTP/Exportin-5 complex [15]. Ran is a GTPase of the Ras protein superfamily. Exportin-5, like other karyopherin nucleocytoplasmic transporters, requires the aid of Ran-GTP to export contents from the nucleus into the cytoplasm [20,21]. Once inside the cytoplasm, pre-miRNA is processed into an miRNA duplex with the removal of its terminal loop and unwound by the RNase III enzyme Dicer [15,22]. At this point, single-stranded mature miRNA joins with the RNA-inducing silencing complex (RISC) to target the mRNA of interest. The RISC complex consists mainly of Dicer, TRBP, and Argonaute2 (Ago2) [23]. Ago2 is the catalytic center of the RISC and is required for strand selection and coordination of target gene silencing events [24]. Finally, the miRNA-induced silencing complex (miRISC) controls gene silencing by cleaving and degrading target mRNA, or by causing translational repression.

### 3.2. Noncanonical Pathways 

There are several noncanonical pathways of miRNA biogenesis. These alternate pathways bypass some of the components normally involved in the canonical pathway. For example, noncanonical miRNA biogenesis may be categorized into Drosha/DGCR8-independent or Dicer-independent pathways [16]. One example of the Drosha/DCGR8-independent pathway involves mirtrons which are pre-miRNAs formed by the splicing of introns and debranching enzymes in the nucleus. They are exported from the nucleus by Exportin-5 and further processed by Dicer and the RISC in the cytoplasm. An example of a Dicer-independent pathway involves the short hairpin RNA (shRNA) processing by Drosha in the nucleus. It is then exported for further processing by Ago2 and the miRISC in the cytoplasm. Recall, Argonaute proteins are a group of proteins that aid in the incorporation of miRNAs with the RISC complex in both the canonical and noncanonical pathways [24,25]. These and other noncanonical pathways are further reviewed in [26]. 

## 4. Dysregulation of miRNAs in Cancer

A role for miRNAs in the development and progression of cancer is well established. In normal tissue, as regulators of gene expression, miRNAs aid in maintaining proper cellular homeostasis. However, in most cancers, miRNAs have been found to be greatly dysregulated [16,27,28,29,30,31]. Alteration in the level of miRNA expression can occur at all stages of tumorigenesis including tumor initiation, progression and metastasis. Specific tumor types can also show a distinct miRNA signature which distinguishes them from their normal tissue of origin and from other cancer types. Some of the consequences of altered miRNA expression that contribute to cancer progression are dysregulated cell growth [31], cell motility in tumorigenesis [32], and/or alteration in hormonal stress response [33]. Dysregulation of miRNAs has been shown to lead to tissue changes that are cardinal features of malignant transformation, including sustained proliferation, evasion of tumor suppression, avoidance of apoptosis, activation of invasion and metastasis, and induction of angiogenesis and drug resistance [27,28]. By causing these changes, miRNAs can act like either oncogenes or tumor suppressors [29]. Although abnormal expression of miRNAs in cancer cells is a widely accepted phenomenon, the causes of this dysregulation are still not yet fully understood. Below, we discuss how deregulation of miRNAs is associated with: (i) defects in miRNA biogenesis with a focus on how *Let-7* affects protein synthesis, and (ii) epigenetic alterations with a focus on aberrant DNA methylation.

### 4.1. Defective Biogenesis 

Defects in miRNA biogenesis itself is one of the mechanistic ways in which miRNAs can lead to cancer progression as both the canonical and noncanonical pathways involve several intermediate factors that are tightly regulated. For example, Dicer protein expression was inhibited in multiple cells lines as a result of reactive oxygen species, phorbol esters, and *Ras* oncogene stress responses [33]. Low levels of dicer expression and functionality are linked to the development and poor survival prognosis of patients with the following cancer types: breast, lung, liver, bladder, and colorectal [34]. Overexpression of Ago1 and Ago2, Dicer, and Drosha were observed in ovarian cancer [35]. Exportin-5 was found to play an oncogenic role in CRC, where high expression levels associated with the worst clinicopathology and poor CRC patient survival [36]. 

### 4.2. Discovery of the LET-7 Family

The identification of the first two miRNAs in *C. elegans*, *lin-4* and *let-7*, led to the discovery of miRNAs in other species and how they are evolutionarily conserved. Indeed, *LET-7* was discovered as the first known human miRNA. Studies on *LET-7* and *LET-7* family members provided the first insight into how miRNAs affect molecular processes in the human body and how its dysregulation causes disease. In fact, *LET-7* is widely known as a tumor suppressor and when under-expressed is associated with poor prognosis in at least 20 different cancers [37]. As tumor suppressors, the *Let-7* family (12 members), downregulates oncogenes such as *c-MYC*, *k-RAS*, *Cyclin D1* and others [38]. In mammals, the biogenesis of *Let-7* is inhibited by the highly conserved RNA binding proteins- *Lin28A* and *Lin28B*. In turn, *Let-7* binds to the 3′ UTR of Lin28 mRNAs to inhibit their expression. In this way, the *LIN28*/*Let-7* pathway operates in a double negative feedback loop and overexpression of *LIN28A* or *LIN28B* is correlated with cancer progression [39] in several different cancer types including liver [40], breast [41], and lung [42]. Balzeau et al. reported on the clinical relevance of *LIN28A/LIN28B* in a table that lists the aforementioned cancers and others, which highlights the impact of the *LIN28/Let-7* pathway in cancer development [43]. 

### 4.3. DNA Methylation and miRNA Expression 

Epigenetic changes due to DNA methylation are known to lead to miRNA dysfunction and contribute to cancer development [15,44]. Indeed, the process of DNA methylation has been shown to occur in unique miRNAs in various cancer types such as oral cancer [45], breast cancer [46], and glioblastoma [47]. DNA methylation affects miRNA expression by silencing or promoting gene transcription. An emerging hallmark of cancer is CpG island hypermethylation of miRNAs that function as tumor suppressors [44]. An example of this was seen in a study evaluating methylation of genes known to be dysregulated in CRC, including *adenomatous polyposis coli* (*APC*)*,* where aberrant hypermethylation was observed in the promoter sequence of *APC* [48]. 

Since miRNAs were implicated in the promotion and suppression of metastasis in breast cancer [49,50,51], Lujambio et al. set out to determine if a unique miRNA hypermethylation profile characteristic of metastasis could be identified [44]. The following miRNAs: *miR-9-1*, *miR-9-2*, *miR-9-3*, *miR-34b*, *miR-34c*, *miR-148a* were found to undergo silencing from hypermethylation which induced metastasis. Although each of the aforementioned miRNAs had varying degrees of hypermethylation, in CRC, *miR-9-3* had the highest prevalence at 84% followed by *miR-148a* (39%), *miR-34b/c* (35%), mir-9-1 (32%), and mir-9-2 (16%) (Table S3 in [44]). In contrast, the frequency of miRNA hypermethylation was roughly equal among lung cancers, ranging from 52–56%. In melanoma, *miR-9-2* hypermethylation was the most prevalent (64%). Breast as well as head and neck tumors were also profiled, but the most notable was that seen for *miR-9-2* in malignant colon tissues. In terms of normal tissue, the miRNAs were not significantly methylated which indicates that hypermethylation of CpG islands is largely correlated with cancer [44].

Alternatively, there are also cases of DNA hypomethylation that affect the control of miRNA expression in cancer as well. For example, hypomethylation of CpG islands just upstream of *miR-196b* led to its overexpression and enhanced cell invasion and migration in oral squamous cell carcinoma [45]. The silencing of *miR-196b* implicated it as having a role in cell invasion and metastasis [45]. Interestingly, there is some level of duality in terms of miRNA function, wherein a particular miRNA can be tumor promoting in one cancer and tumor suppressing in another. For example, *miR-196b* hypomethylation causes its overexpression and promotes progression of oral squamous cell cancer, and its hypermethylation causes tumor suppressor activities in lung carcinoma [52].

The modification of DNA that occurs in methylation is mediated by DNA methyltransferases (DNMTs) [53]. One example of how these enzymes facilitate cancer progression is by transcriptional repression [54]. In glioblastoma, overexpression of DNMTs is associated with hypermethylation of tumor suppressor genes [55], and the *miR-296-5p* tumor suppressive ability (downregulation of stemness genes) was reversed by DNMT-dependent hypermethylation [47]. On the other hand, DNMT3b was found to directly repress stemness genes in breast cancer [46]. The authors found that *miR-221* acts as a suppressor and downregulates *DNMT3b* expression by binding to its 3′ untranslated region (3′ UTR). In doing so, the downregulation of DNMT3b by *miR-221* aids in breast tumorigenesis by alleviating its repression of stemness [46]. Thus, the functional variability in DNA methylation and the role of its targeted miRNAs is evident in the dysregulated gene expression in different cancer types.

## 5. miRNA Clusters and Their Role in Tumor Development

Since their discovery, genome-wide studies identified several hundred evolutionarily conserved miRNAs essential for diverse biological processes through their regulation of gene expression. A recent study estimated there are possibly 2300 mature miRNAs that exists in the human genome [56]; an update to the miRBase database report accounted for 2656 around the same time [57]. miRNAs are typically encoded in between genes (intergenic), but some are encoded within introns (intragenic) of pre-miRNAs or noncoding RNAs [58]. In this way, it is quite reasonable to understand how miRNAs may be grouped or clustered together. This phenomenon is also evolutionarily conserved, and in humans, approximately 40% of miRNA genes are clustered [58]. Large clusters may be transcribed as a single polycistronic transcript encoding for multiple miRNAs with similar expression patterns and either similar or different functions. Polycistronic miRNAs have also been shown to regulate each other and when disrupted, can result in developmental defects [59]. 

Genomic redundancy, where homologous polycistrons are found on multiple chromosomes, implicates a common function. An example is shown where *miR-99a/100, let7,* and *miR125b* converge to block the TGFβ pathway and upregulate Wnt signaling in the disruption of hematopoietic stem cell homeostasis in acute megakaryoblastic leukemia (AMKL) [60]. However, opposing functions of individual members of the aforementioned polycistron, *miR-99a/100* [61] and *LET-7* [62] were also identified. Therefore, understanding how clusters (and their individual miRNAs) function is important as they may prove useful in therapeutic targeting [63]. Altogether, given the functional variability of individual miRNAs and clusters, it is of no surprise that dysregulation can result in different disease types. In this section, we will identify and review current literature on two clusters and their individual miRNA members implicated in colon and other cancers.

### 5.1. miR-17-92 Cluster

*MiR-17-92* is one of the most characterized clusters, known as an oncogene, but responsible for a variety of disease pathologies. This cluster regulates biological processes such as cell proliferation, apoptosis, metabolism, metastasis, and tumorigenesis. Its overexpression is well-established in a variety of tumor cells and cancer types including colon [64] and lung [65,66]. The *miR-17-92* cluster is polycistronic, located on human chromosome 13, and encodes for 7 individual mature miRNAs *miR-17-3p*, *miR-17-5p*, *miR-18a*, *miR-19a*, *miR-19b-1*, *miR-20a* and *miR-92a-1* [4]. Various roles of the miR-17-92 cluster in neurological, heart disease, and bone development are reviewed in [4]. In tumorigenesis, the cluster as a whole and/or by its individual members targets either of three major pathways that regulate cell proliferation and/or apoptosis: JAK/STAT, PI3K/AKT/mTOR, and PTEN [4]. The functional variety of target genes and subsequent pathways they regulate may explain the cell- and context-dependent role of *miR-17-92*. 

Elevated levels of *miR-17-92* were associated with tumorigenesis [67,68,69] and poor survival rates in CRC [70]. A direct link between oncogenic *miR-17-92* and tumor suppressor *APC* was discovered where β-catenin upregulated expression of miR-17-92 in *APC* mutant cells, and *wt-APC* caused degradation of β-catenin and reduced *miR-17-92* [71]. *miR-17-92* downregulated multiple components of TGFβ signaling to evade cell cycle arrest and/or apoptosis in neuroblastoma [72], and upregulation of *miR-17-92* by c-Myc caused downregulation of TGFβ signaling pathway components to stimulate angiogenesis and tumor growth [73]. Conversely, an anti-angiogenic role was identified in CRC where *miR-17-92* downregulated angiogenic inducing genes: *TGFBR2*, *HIF1α*, and *VEGFA* [74], and a tumor suppressive role was identified in prostate cancer [75].

Individual miRNAs within a cluster can have differing functions from the group and/or from each other. For example, *miR-18a* was correlated with *APC* mutations and is highly expressed in colon tumors [76]. *miR-19a* and *miR-19b* are essential for oncogenic activity of the entire *miR-17-92* cluster by reducing PTEN expression and its tumor suppressor activity [77]. *miR-20* activates cyclin-dependent kinase inhibitor 1A/p21 (CDKN1A/p21), which negatively regulates TGFβ, and thus prevents its antiproliferative effect in CRC [4]. In addition, *miR-17-5p* and *miR-20* reduced expression of TGFβ-receptor type II and *miR-18* limited expression of Smad4 [73]. 

### 5.2. miR-92a Family

While *miR-92a* is part of the *mir-17-92 cluster*, *miR-92a* is also a member of a conserved miRNA family: *miR-92a-1*, *miR-92a-2*, *miR-363* and *miR-25.* The *miR-92a* family arose from three different paralog clusters *miR-17-92*, *miR-106a-363*, and *miR-106b-25* that occurred during evolution. Studies on *miR-92a* reveal it plays a key role in regulating organ development and tumorigenesis, and is overexpressed in several tumors including: colon, prostate, lung, pancreatic, stomach, pancreas, and others [78]. A recent study discovered that overexpression of *miR-92a* caused increased proliferation of glioma cells by targeting the KLF4/AKT/mTOR pathway [79]. Several mechanisms involving the downregulation of tumor suppressor and apoptosis genes and upregulation of cell proliferation have been implicated in the tumorigenic role of *miR-92a* in CRC [80,81,82,83,84]. Furthermore, high expression levels of *miR-92a* correlated with tumor metastasis and poor prognosis [85,86,87] and it is demonstrated to be a novel diagnostic biomarker [88] in CRC. It is important to note, an opposing role for *miR-92a* was observed in breast cancer, where downregulation of *miR-92a* was associated with aggressive features and increased macrophage infiltration [89].

### 5.3. miR-23b-27b-24 Cluster

The *miR-23b-27b-24* cluster is not as well-characterized as the miR-17-92 cluster and its role in cancer has been controversial and understudied. Most studies focus instead on the function of its individual miRs: *miR-23a/b*, *miR-27a/b*, and *miR-24-1/2*. Nevertheless, two paralogs exist in humans, the *miR-23b-27b-24-1* cluster, which is encoded within an intron on the *C9orf3* gene located on chromosome 9, and *miR-23a-27a-24-2* located on chromosome 19. The two paralogs are predicted to have the same gene targets, but so far only ~15% of the predicted shared targets that have been validated as discussed in [90]. 

In a recent study, the *miR-23b/27b/24* cluster has been found to aid in the process of cell migration in a subset of cells with high migration capacity in several CRC cell lines [91]. This study is interesting because it addresses the issue of heterogeneity of tumor cells. In this way, *miR-23b/27b/24* may be considered tumor progressive as cell migration is required for tumor invasion, metastasis, and epithelial to mesenchymal transition (EMT) of cells. The authors also examined the individual miRNAs of the cluster and determined that only *miR-23b* and *miR-27b* were effective in regulating cell migration by direct inhibition of FOXP2 (*miR-24* did not). FOXP2 was recently implicated as a suppressor of metastasis and identified as a possible prognostic marker in breast cancer [92]. Downregulation of FOXP2 by the *miR-23b-27b-24-1* cluster and two of its individual members warrants the further investigation of clusters as this approach may have greater therapeutic value than for individual miRNAs. In line with this finding, the cluster, but not its individual members, conferred resistance to oxaliplatin by modulating EMT in CRC [93]. 

The miR clusters may display oncogenic or tumor suppressive roles depending on their context. The tumor suppressive ability of *miR-23b/27b/24-1* was demonstrated in prostate cancer (PCa). Expression of *miR-23b-27b-24-1* was reduced in clinical PCa tissue specimens, and gain of function studies resulted in reduced cell proliferation, migration, and invasion in two PCa cell lines [94]. In non-small cell lung cancer (NSCLC), *miR-23b-27b-24-1* is downregulated by altered expression of platelet-derived growth factor (PDGF). Abnormal expression of PDGF receptors is a clinical prognostic marker for the worst cases of NSCLC. Induced expression of *miR-23b-27b-24-1* increased drug sensitivity and reduced invasiveness of NSCLC cells by silencing gene members of the oncogenic NF-κB and KRAS pathways [95], also identifying a tumor suppressive role for this cluster. 

Individual members of *miR-23b-27b-24-1* also display varying roles in different cancer types. In breast cancer, *miR-23b* and *miR-27b* was shown to promote oncogenesis [96]. *miR-23b* and *miR-27b* are highly expressed in vascularized tissues and epithelial cells. *miR-23b* and *miR-27b* also promote angiogenesis by downregulating anti-angiogenic proteins, and their inhibition repressed angiogenesis [97]. However, *miR-27b* is downregulated in colon cancer by c-SRC and KRAS, suggesting a tumor suppressive role. This role was confirmed by finding that *miR-23b* downregulates ARFGEF1 and paxillin expression to prevent tumor growth and invasion of colon cancer [98]. Individually, *miR-23b* has a role in a variety of cellular processes including cell differentiation, immunity, and cancer progression. This single miRNA is pleiotropic, exhibiting gene regulation of a variety of cellular processes in the human body [99]. Thus, its dysregulation can lead to a variety of disease pathologies, including tumor progression or suppression. *miR-23b* expression is downregulated in CRC, and several targets: TGFβR2, uPA, MAP3K1, PAK2, FZD7 [100] and PDE7A [101] have been identified, implicating a tumor suppressive role. A genome-wide study revealed the ability of *miR-23b* to subdue tumor metastasis [99]. However, the role of *miR-23b* in metastasis was more recently found to be context-dependent, as it cooperates with BTBD7 to promote metastasis in CRC [102]. We will discuss how *miR-92a* and *miR-23b* contribute to the development of different populations of stem cells in CRC below, but first we will discuss cancer stem cell theory.

## 6. Cancer Stem Cell Theory and the Role of miRNAs

In CRC development, SC overpopulation has been found to be a driver of tumor initiation and progression by us [103,104,105,106,107] and others [108,109,110]. Since miRNAs have a critical role in regulating SCs during development, and altered expression of miRNAs occurs in various cancers, our focus has been to study whether miRNA dysregulation is involved in the SC origin of CRC. We conjecture that different CSC populations are generated by the dysregulation of different miRNAs. We will begin by discussing one of the first proteins (CD44) identified as a SC marker and how it provided an explanation for the role of miRNA dysregulation in carcinogenesis. Then we will explore how miRNAs might regulate expression of SC genes and lead to CSC populations in CRC.

### 6.1. CD44 

The hyaluronate receptor/P-glycoprotein 1 (CD44) is a transmembrane protein [111], which has been widely studied as a SC marker for various cancer types. The function of CD44 involves interactions with different ligands, especially hyaluronidase [112]. In breast cancer and cervical cancer, CD44 interacts with the beta-catenin and Akt pathways which promote epithelial to mesenchymal transition often observed in carcinogenesis [113]. Recent studies on human CRC show that CD44, when coupled with LGR5, can efficiently identify cancer SCs [114]. CD44 has also been looked at with another biomarker called aldehyde dehydrogenase 1 (ALDH1). Together CD44 and prominin-1/AC133 (CD133) along with ALDH1 were studied as biomarkers that can track stem cell overpopulation during CRC formation [111]. 

CD44 is an example of one of the many biomarkers that can explain how miRNA dysregulation is correlated with carcinogenesis. Studies on CD44 expression in CRC showed that modulating Dicer expression led to changes in CSCs and miRNAs [34]. For example, impairment of DICER1 function led to an upregulation of CD44, Sox9, Sox2, Nanog, and Lgr5. In cells with impaired Dicer function, a reduction of multiple miRNAs that target CD44 and the Wnt/beta-catenin pathway mediated the upregulation of CD44 expression. Impaired DICER1 was also linked to the generation of subpopulations of CRC cells with enhanced SC features and plasticity displayed by the interconversion between heterogeneous CRC cell subtypes. Tumor heterogeneity and cellular plasticity can enhance the progression of CRCs as tumor cells acquire an ability to withstand therapy-induced selection pressure [115]. A consequence of dysregulated DICER1 function and deregulation of miRNAs that promotes stemness is an enhanced ability to initiate tumors and metastasis [34].

### 6.2. Other SC Markers 

The role of aberrant miRNA expression in CSCs in cancer has recently been the subject of several reviews [116,117,118,119]. We will provide an update by discussing miRNAs and the SC genes they are predicted to target in CRC. First, we provide a brief review of the SC genes (ALDH, CD166, LGR5, LRIG1, BMI1) that are the focus of this paper. Aldehyde dehydrogenase (ALDH) is a marker discovered by us and others that identifies SCs in normal and malignant colonic tissues and can track SC overpopulation during tumor growth [111,120,121]. ALDH1 is the enzyme responsible for metabolizing retinal into retinoic acid and is known to play an important role in cellular differentiation [122]. ALDH1 has also been found to be a SC marker for many other human cancers such as breast, lung, and ovary [123,124]. Next, CD166 (ALCAM) was identified and these CD166+ cells were shown to possess tumor-initiating ability [125]. Then, BMI1, LGR5, and LRIG1 were identified as SC markers in humans as well as mice [126,127,128]. The LGR5 transmembrane protein is expressed in proliferating SCs and observed to be responsible for expansion of the SC compartment and adenoma formation in mice [109,127]. BMI1, a member of the polycomb protein family, is a hematopoietic SC marker that is over-expressed in various tumor types [127,129,130,131]. LRIG1 is a pan-ERBB negative regulator and promotes SC quiescence in the epidermis and the intestinal tract [128,132,133]. These markers and several others have been used to identify and isolate different CSC populations from CRCs (reviewed in [107,134,135,136,137]). However, there is a paucity of data to understand how these different CSC populations develop and are maintained in CRCs. We explore the possible role of miRNAs as is presented in this paper.

## 7. miRNAs Implicated in Development of CRC Stem Cell Populations

Our recent studies have investigated how miRNAs might regulate expression of SC genes and lead to the development of the different CSC populations [136] and their overpopulation in CRC. Indeed, an investigation of the literature shows that many miRNAs have been reported to target SC genes or genes involved in regulating SC properties (Table S1). Moreover, using miRNA expression profiling of isolated normal and malignant human colonic SCs [138,139], we showed that *miR-23b* and *miR-92a* are regulators of the *LGR5* and *LRIG1* SC genes, respectively. Findings from these two published studies and from our review of TCGA study results are described further below.

### 7.1. miRNA23b

Our study designed to analyze miRNA expression in the colonic SC niche using micro-dissected human colonic crypts identified a set of miRNAs that distinguishes malignant from normal colonic epithelium [138]. Notably, *miR-23b*, which was increased in CRC, was predicted to target the *LGR5* SC gene. We also showed that *miR-23b* regulates CSC phenotypes globally at the level of proliferation, cell-cycle, self-renewal, EMT, invasion, and resistance to 5-FU. *miR-23b* decreased LGR5 expression and increased the number of ALDH+ CSCs. We confirmed that levels of *LGR5* and *miR-23b* are inversely correlated (*p* < 0.05) in ALDH+ CSCs, and that distinct populations of LGR5+ and ALDH+ CSCs exist. Overall, our study defined a critical function for *miR-23b*, which, by targeting *LGR5*, contributes to overpopulation of ALDH+ CSCs and CRC.

### 7.2. miRNA92a

We also took an independent approach to identify miRNAs in purified human colonic SCs [139], which was based on a more extensive screen involving miRNA expression profiling of ALDH+ SCs isolated from fresh normal colonic epithelium and CRC tissue. This was done to identify additional miRNAs involved in the SC origin of CRC. We used Nanostring expression profiling to analyze miRNAs (*n* = 800) in SCs purified from fresh normal and malignant colonic tissues. We found: (1) a unique miRNA signature that differentiated ALDH+ CSCs from ALDH+ normal SCs; (2) expression of four miRNAs (*miR-200c*, *miR-92a*, *miR-20a*, *miR-93*) were upregulated in CRC CSCs compared to normal colonic SCs; (3) *miR-92a* was upregulated in ALDH+ HT29 CRC CSCs; (4) *miR-92a* targeted the 3′UTR of *LRIG1*; and (5) *miR-92a* modulated proliferation of HT29 CRC cells. Thus, our findings indicated that overexpression of *miR-92a* also contributes to the SC origin of CRC.

## 8. miRNAs Predicted to Target CRC Stem Cell Genes

**Figure 2:**
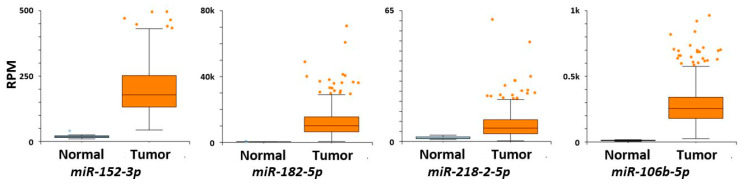
Box chart graphs of miRNA levels in normal colon (left box) compared to CRC (right box) (Y axis = normalized RNA-seq data (RPM)).

**Figure 3:**
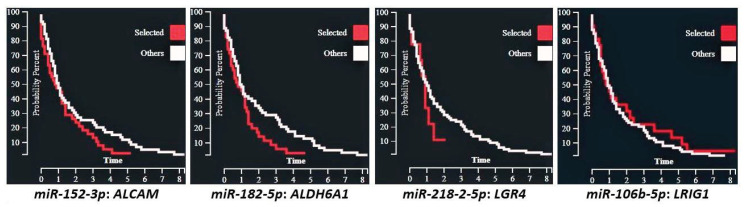
Kaplan−Meyer CRC survival curves for miRNA cases selected by their SC target gene versus all other cases, X-axis = years.

A review of miRNAs that target SC genes in normal versus cancerous colonic tissues was done herein using the two sets of miRNAs (*n* = 164 total miRNAs) from our previous published studies on differentially expressed miRNAs in colon CSCs [138,139]. A literature review was then done to see whether these miRNAs are reported to target SC genes in normal colon vs. CRC. The miRNAs from these two studies on colonic SCs [138,139] were cross referenced with miRNAs reported in the TCGA database for: (a) miRNA expression in normal vs CRC tissue, (b) prediction of target SC genes, (c) whether the expression of a miRNA inversely correlates with the mRNA levels of the target gene, and (d) correlation of the expression of miRNAs with CRC patient survival and other clinical parameters. The results (Table 1) showed that 53 miRNAs are predicted to target the SC genes analyzed in the study and 39 of them showed differences (*p* < 0.01; Table S2) in expression between normal and CRC tissues (see examples in Figure 2). Of the miRNAs, 13 are predicted to target more than one SC gene. We further classified these miRNAs that are predicted to target SC genes based on the miRNA clusters that they are part of along with the other members of the cluster, including the changes in miRNA expression between normal colon vs. colon cancer (Table S2). The specific miRNA isoform (isomiR) that is predicted to target the SC genes is also given in Table S2. Figure 2 gives some examples of isomiR expression in normal colon compared to CRC. Figure 3 shows examples of survival curves for CRC cases based on expression of these same isomiRs selected by inverse mRNA expression of target SC genes. 

## 9. Discussion

Our previous and ongoing studies indicate that miRNAs target SC genes in normal colonic epithelium, and when dysregulated, these miRNAs play a role in the SC origin of colon carcinomas. Indeed, we previously reported that *miR-23b* and *miR-92a* are regulators of the *LGR5* and *LRIG1* SC genes, respectively. Our review of data reported in the TCGA study indicates that other miRNAs also target SC genes and many miRNAs show differences in expression between normal and CRC tissues. Several of these miRNAs are members of the same miRNA cluster and other members within the cluster are also predicted to target SC genes. Moreover, the expression of a number of the miRNAs inversely correlates with the mRNA levels of the target gene, and expression of the miRNAs correlates with CRC patient survival and other clinical parameters. Several points bear further discussion.

### 9.1. What Is the Role of Isomers in the Development of Different CSC Populations?

We also considered isomiRs based on differences in their SC target prediction because recent findings show that variations in miRNA sequences are known to display differences in expression in various types of tissues and cancers [140,141]. Indeed, it has been found that isomiR expression profiles distinguish different breast cancer subtypes [142]. Our findings herein show that different isomiRs from the same reference miRNA can differ in their ability to target a given SC gene. We also found that some 3*p* and 5*p* isomiRs differ with regard to being upregulated or downregulated in CRC versus normal colon. In addition, the association of a miRNA with CRC patient survival often correlated with just one or the other of the isomiRs from the same reference miRNA. These findings indicate that each isomiR has a distinct impact on the transcriptome of colon CSCs.

### 9.2. How Might miRNA Expression Lead to the Development of Different CSC Populations in CRC?

Our findings show that specific miRNAs are selectively expressed in different human colon CSC populations and some of these miRNAs target known SC genes. For example, we found that levels of LGR5 and *miR-23b* are inversely correlated in ALDH+ colon CSCs, and that distinct subpopulations of LGR5+ and ALDH+ CSCs exist [138]. Moreover, we found that transfection of human CRC cells with *miR-23b* precursor increased the proportion of ALDH+ cells and transfection with the *miR-23b* anti-miRNA had the opposite effect. We also found that *miR-92a*, which targets the 3′UTR of *LRIG1*, is upregulated in ALDH+ colon CSCs and *miR-92a* modulates CRC cell proliferation [139]. Further review of data from the TCGA database was done herein to identify miRNAs that target SC genes in normal versus cancerous colonic tissues using a set of miRNAs from our previous studies on miRNAs in CRC. These results reveal that many miRNAs predicted to target SC genes inversely correlate with levels of SC gene expression and CRC patient survival. Taken together, these results indicate that miRNAs that target SC genes in a given CSC population suppress expression of specific SC genes expressed in other CSC populations (and vice versa). Our results indicate that miRNAs, when dysregulated, play a major role in the development of different populations of CSCs and SC overpopulation in CRC. 

## 10. Conclusions

In this manuscript, we have reviewed miRNA expression in colon CSCs in order to identify mechanisms that can explain the development of different CSC populations in CRC. Our previous miRNA expression profiling of isolated normal and malignant human colonic SCs showed that *miR-23b* and *miR-92a* are regulators of the *LGR5* and *LRIG1* SC marker genes, respectively. Our further online investigation of these miRNAs indicates that many miRNAs predicted to target SC genes regulate SC gene expression and correlate with CRC patient survival. Thus, we conjecture that miRNAs contribute to regulation of SC genes and genes involved in regulation of SCs and, when dysregulated, contribute to the emergence of different CSC populations and SC origin of CRC.

Continued research on mechanisms that explain how miRNAs regulate normal colon SCs and how dysregulation of miRNAs in CSCs drives CRC growth should provide insight into how new SC-targeted therapies might be designed for CRC. For example, miRNA studies could provide insight into how different CSC subpopulations arise in CRC and advance our understanding of how tumor heterogeneity occurs. It could also provide clues as to how to eliminate CSCs and improve the efficacy of anticancer therapies. For example, novel, effective therapeutics could be designed to: (1) directly target miRNAs identified as vital to specific CSC populations; and/or (2) modulate CSC composition in order to therapeutically sensitize tumors. Indeed, an emerging field is focusing on combining miRNAs with other drugs to modulate dysregulated miRNAs and sensitize cancers to conventional or immunotherapy agents [143,144,145,146,147]. Thus, the significance of this deliberation is that discovering how to modulate the function of miRNAs may provide a mechanism to reverse changes in miRNA expression patterns and to therapeutically target CSCs. Overall, discovery of miRNAs that regulate CSCs offers great potential for improving cancer research and clinical oncology practice paradigms. 

## Figures and Tables

**Table 1:** Survey of miRNAs (*n* = 216) aberrantly expressed in CRCs that target stem cell genes *.

ALCAM	ALDH Isoforms	BMI1	LGR Isoforms	LRIG Isoforms	
*mir-142-5p*	*mir-182-5p*	*miR-106b*	*mir-142*	*mir-20a-5p*	*mir-218-2-5p*
*mir-148a-3p*	*mir-23a-3p*	*miR-107*	*mir-23b*	*mir-023a-3p*	*mir-92a-1*
*mir-148b-3p*	*mir-23b-3p*	*miR-153-2*	*mir-218-2-5p*	*mir-023b-3p*	*mir-93-5p*
*mir-152-3p*	*mir-27a-3p*	*miR-154-3p*	*let-7a-1-5p*	*mir-106a-5p*	*let-7a-1-5p*
*mir-9-1-5p*	*mir-27b-3p*	*miR-208b*	*let-7a-2-5p*	*mir-106b-5p*	*let-7a-2-5p*
*mir-9-2-5p*	*mir-31-5p*	*miR-218-2-5p*	*let-7a-3-5p*	*mir-129-1-p5*	*let-7a-3-5p*
		*miR-27a*	*let-7b-5p*	*mir-129-2-p5*	*let-7b-5p*
		*miR-302c*	*let-7c-5p*	*mir-130b-3p*	*let-7c-5p*
		*miR-033b*	*let-7d-5p*	*mir-16-1-5p*	*let-7d-5p*
		*miR-539-3p*	*let-7e-5p*	*mir-16-2-5p*	*let-7e-5p*
		*miR-548j*	*let-7f-1-5p*	*mir-17-5*	*let-7f-1-5p*
		*miR-548h*	*let-7f-2-5p*	*mir-200c*	*let-7f-2-5p*
		*let-7g*	*let-7g-5p*	*mir-208b*	*let-7g-5p*

* Survey of the TCGA database done using MIR-TV tool (http://mirtv.ibms.sinica.edu.tw/). Note: isomiRs refer to the mature miRNA products that are produced from a given arm (5p from the 5’ arm of the precursor or 3p from the 3’ arm of the precursor) of a given miRNA precursor.

## Data Availability

The sources of the publicly archived datasets analyzed are: miRBase. Available online: http://www.mirbase.org/ (accessed on 18 December 2020); TargetScanHuman. TargetScanHuman 7.2. Available online: http://www.targetscan.org/vert_72/ (accessed on 21 December 2020); GeneCards. Human Genes | Gene Database | Gene Search. Available online: https://www.genecards.org/ (accessed on 21 December 2020).

## Supplementary Materials

The following are available online at https://www.mdpi.com/1422-0067/22/3/1424/s1. Table S1: miRNAs predicted to target SC genes or genes involved in regulating SC functions. Table S2: Classification of miRNAs based on the miRNA clusters and isomiR.
