# Supplementary material for: The Role of miRNAs, miRNA Clusters, and isomiRs in Development of Cancer Stem Cell Populations in Colorectal Cancer
Source: Int J Mol Sci. 2021 Jan 31;22(3):1424. doi: 10.3390/ijms22031424 (PMC7867000; doi:10.3390/ijms22031424)
Supplement: Supplementary file 1 [file ijms-22-01424-s001.pdf]

Supplementary Material

Table S1. miRNAs predicted to target SC genes or genes involved in regulating SC functions				
**miRNA**	**Targets**	**Disease/Cancer**	**Role**	**Reference**
miR-17-92 cluster	APC gene	colon	cancer progression	71
miR-17-92 cluster	TGF-Beta Pathway ↓	neuroblastoma	cancer progression	72
miR-17-92 cluster	C-Myc	colon	inhibits TGF-Beta and causes angiogenesis	73
miR-17-92 cluster	FGD4/LIMK1 /cyclin D1/SSH1	prostate	↓ expression in Rho-GTPase pathway	75
miR-17-92 cluster	PTEN/RB2	lung	cluster overexpression in cancer	65
miR-17-92 cluster	PTEN	neurological diseases	regulation of PTEN also affects degradation of neurons	4
miR-17-92 cluster	PTEN	cardiac disease	cluster overexpression = cardiomyocyte proliferation	4
miR-23b/27b/24 cluster	PDGF	lung	affects KRAS and NF-Kb pathways	95
miR-23b/27b/24 cluster	FOXP2 ↓	colon	downregulates FOXP2	91
miR-23b/27b/24 cluster		breast	oncogene	96
miR-23b/27b/24 cluster	ARGEF1	colon	tumor suppression	98
miR-23b/27b/24 cluster	anti-angiogenic proteins ↓	Choroidal neovascularization	angiogenesis	97
miR-23b/27b/24 cluster	5-Fluorouracil and Oxaliplatin Chemotherapy	colon	resist oxaliplatin treatment, EMT in cancer	93
miR-17 family		bone development	regulates bone metabolism and formation	4

miR-18a	APC gene	colon	highly expressed in tumors	76
miR-19	PTEN	B-cell lymphomas	KD of PTEN aids in cancer progression	77
miR-20	CDKN1A/p21	colon	tumorigenesis	4
miR-92a	KLF4 and AKT/mTOR pathway	glioma	cancer progression	79
miR-92a		breast cancer	↓ miRNA expression in breast cancer cells	89
miR-92a	Dkk-3 gene	colon	tumorigenesis	80
miR-92a	Bim ↓	colon	cancer progression	81
miR-92a	RECK ↓	colon	cancer progression	82
miR-92a	KLF4/GSK3-Beta/DKK-3	colon	IL-6/STAT3 pathway ↑ miR-92a/Wnt/Beta-Catenin signaling	83
miR-92a	BMP5 ↓	colon	tumorigenesis	84
miR-92a	LRIG1 ↓	colon	downregulates LRIG1	140
miR-23b	FOXP2	colon	pro-metastasis	91
miR-23b	TGFbR2 ↓	colon	cancer progression	100
miR-23b	uPA ↓	colon	cancer progression	100
miR-23b	MAP3K1 ↓	colon	cancer progression	100
miR-23b	PAK2 ↓	colon	cancer progression	100
miR-23b	FZD7 ↓	colon	cancer progression	100
miR-23b	BTBD7	colon	cancer metastasis	102
miR-23b	PDE7A ↓	colon	cancer progression	101
miR-23b	LGR5 ↓	colon	downregulates LGR5	139
miR-27b	GOLM1 ↓	castrate resistant prostate	cancer progression	94
miR-27b	FOXP2	colon	pro-metastasis	91
miR-24	FOXP2	colon	anti-metastasis	91
miR-9-2	CpG	melanoma	DNA Hypermethylation	44
miR-9-3	CpG	colon	DNA Hypermethylation	44
miR-148a	CpG	colon	DNA Hypermethylation	44
miR-196b	CpG	oral squamous	DNA Hypomethylation	45
miR-196b	FOS gene	lung	DNA Hypermethylation	52
miR-221	DNMT3b ↓	breast	cancer progression	46

miR-296-5p	HMGA1	glioblastoma	inhibits tumorigenesis	47

Table S2. Classification of miRNAs based on the miRNA clusters and isomiR

**Chromosome**	**Cluster**	**Predicted SC Gene Targets**	**TCGA: Normal vs CRC**
Chr3	[1]hsa-mir-15b-5p broadly conserved	none	↓ $p < 0.01$
	hsa-mir-15b-3p	ALDH1B1, 3B1, 7A1, 1A1, BMI1, LGR5, LGR4,	↑ $p< 0.01$
	[1]hsa-mir-16-5p	LRIG1	↑ $p< 0.01$
	[2]hsa-mir-16-2-3p	ALCAM, ALDH1L2, 6A1, 1A2, 7A1, 1A3, 5A1, BMI, LGR5, LGR4, LRIG1, LRIG2	↑ $p< 0.01$
Chr7	[3]hsa-mir-106b-5p Broadly conserved	None	↑ $p< 0.01$
	hsa-mir-106b-3p	LGR4	↓ $p < 0.01$
	[3]hsa-mir-93-5p Broadly conserved	None	NS
	hsa-mir-93 - 3p	ALDH1A2, 5A1, LGR6, LRIG2	NS
	hsa-mir-25-5p	ALDH1B1, 5A1, 1L2, 6A1, LRIG2	NS
	[4]hsa-mir-25-3p	None	↑ $p< 0.01$
Chr7	hsa-mir-183-5p.1	BMI1	↑ $p< 0.01$
	hsa-mir-183-5p.2	ALDH6A1, LRIG1	↑ $p< 0.01$
	hsa-mir-183-3p	ALDH1B1, 1A3, 6A1	NS
	[5]hsa-mir-96-5p	LRIG1	↑ $p< 0.05$
	hsa-mir-96-3p	ALCAM, ALDH1B1, 1L2, LGR4, LGR5, LGR6	NS
	hsa-mir-182-5p	ALDH6A1, LRIG2	↑ $p< 0.01$
	hsa-mir-182-3p	ALDH1A2, 1A3, 5A1, 6A1, LRIG2, LRIG3	NS

**<u>Chr9</u>**	hsa-mir-23b-5p	ALDH18A1, 7A1, 5A1, LGR4, LGR5, LRIG2, LRIG3	↓ $p < 0.05$
	hsa-mir-23b-3p broadly conserved	ALDH1A2, 1L2, LRIG1, LRIG2	↓ $p < 0.01$
	hsa-mir-27b-5p	ALDH1B1, 1A2, BMI1, LRIG2	↑ $p< 0.01$
	hsa-mir-27-3p broadly conserved	ALDH4A1, 1L2, BMI1, LGR4	NS
	hsa-mir-3074-5p	ALCAM, ALDH2, 7A1, 5A1, 9A1, 1L2, BMI1, LRIG2, LRIG3	↓ $p < 0.01$
	hsa-mir-3074-3p	ALDH7A1, 1L2, 6A1, BMI1, LRIG2, LRIG3	NS
	hsa-mir-24-1-5p	ALDH1B1, 1A2, 3A2, 4A1, 7A1, 6A1, LRIG3	↓ $p < 0.01$
	hsa-mir-24-1-3p	ND	↑ $p< 0.01$
**<u>Chr9</u>**	[6]hsa-let-7a-1-5p	None	$p < 0.01$
	[7]hsa-let-7a-1-3p	ALDH1A3, 7A1, 5A1, BMI1, LGR4, LGR5, LRIG3	$p < 0.01$
	[6]hsa-let-7f-1-5p	LGR4	$p < 0.01$
	[7]hsa-let-7f-1-3p	ALCAM, ALDH1L2	NS
	[6]hsa-let-7d-5p	ALDH6A1, LRIG2, LRIG3	NS
	hsa-let-7d-3p	ALDH3A2, LRIG2	$p < 0.01$
Chr11	[8]hsa-mir-100-5p	None	NS
	hsa-mir-100-3p	ALCAM, ALDH1A1, 1B1, 7A1, 1L2. 6A1, LGR4, LRIG2, LRIG3	NS
	hsa-mir-10526	ND	ND
	hsa-let-7a-2-5p	ND	$p < 0.01$
	[9]hsa-let-7a-2-3p	ALDH8A1, 9A1, 1A2, BMI1, LGR4, LRIG1, LRIG3	$p < 0.01$

Chr12	[10]hsa-mir-200c-5p	ALCAM, BMI1, LRIG2, LRIG3	↑ $p < 0.01$
	[11]hsa-mir-200c-3p	None	↓ $p < 0.01$
	hsa-mir-141-5p	ALCAM, ALDH8A1, 18A1, 1L1, LRIG2	↑ $p < 0.01$
	[12]hsa-mir-141-3p	None	↑ $p < 0.01$
**<u>Chr13</u>**	hsa-mir-17-5p broadly conserved	None	↑ $p < 0.01$
	hsa-mir-17-3p	ALDH1A1, 3B2, 5A1, 1A3, 3A2, 16A1, 7A1, 1L2, LGR4, LRIG2	↑ $p < 0.01$
	hsa-mir-18a-5p broadly conserved	ALCAM	NS
	hsa-mir-18a-3p	ALDH3A2, 5A1, 1A2, 6A1, 7A1, LRIG2	↓ $p < 0.05$
	hsa-mir-19a-5p	ALDH1L2, BMI1, LGR4	ND
	hsa-mir-19a-3p broadly conserved	LRIG1, LRIG3	ND
	hsa-mir-20a-5p broadly conserved	LRIG1	↑ $p < 0.01$
	hsa-mir-20a-3p	ALDH1A2, 3B1, 3A2	↑ $p < 0.01$
	hsa-mir-19b-1-5p	ALDH1A2, 8A1, 18A1	↑ $p < 0.01$
	hsa-mir-19b-1-3p	ND	↑ $p < 0.01$
	hsa-mir-92a-1-5p	ALDH3B1, 1B1, 1A2, 6A1	↓ $p < 0.01$
	[4]hsa-mir-92a-1-3p	None	↓ $p < 0.01$
Chr17	hsa-mir-4736-5p	ALDH7A1, 3B2, 1L2, 5A1, 3A2, 6A1, 4A1, LRIG3	NS
	hsa-mir-142-5p	ALCAM	↑ $p < 0.01$

	hsa-mir-142-3p.1	None	↑ $p< 0.01$
	hsa-mir-142-3p.2	ALCAM, ALDH1A3,	↑ $p< 0.01$
Chr17	hsa-mir-152-5p	ALCAM, 1A3, 1A2, 7A1,3A2, 5A1, 6A1, LGR4, LRIG2, LRIG3	NS
	[13]hsa-mir-152-3p	ALCAM	↑ $p< 0.01$
	hsa-mir-10226	ND	ND
Chr19	hsa-mir-23a-5p	ALDH18A1, 7A1, 5A1, LGR4, LGR5, LRIG2, LRIG3	↓ $p < 0.05$
	hsa-mir-23a-3p	ALDH1L1, LRIG1	↑ $p< 0.01$
	hsa-mir-27a-5p	ALDH1B1, 1A2, BMI, LRIG2	↑ $p< 0.01$
	hsa-mir-27a-3p	ALDH4A1, 1L2, BMI, LGR4	↑ $p< 0.01$
	hsa-mir-24-2-5p	1B1, 1A2, 3A2, 4A1, 7A1, 6A1, LRIG3	↑ $p< 0.01$
	hsa-mir-24-2-3p	ND	↑ $p< 0.01$
Chr19	[8]hsa-mir-99b-5p	None	↓ $p < 0.01$
	hsa-mir-99b-3p	ALDH1A3, 7A1, LGR4, LRIG3	↓ $p < 0.01$
	[6]hsa-let-7e-5p	None	$p < 0.01$
	hsa-let-7e-3p	ALDH3A2,	$p < 0.01$
	hsa-mir-125a-5p	None	↓ $p < 0.01$
	hsa-mir-125a-3p	ALDH3B1, 5A1, 3A2, 1L2, LRIG2	↓ $p < 0.05$
Chr21	[8]hsa-mir-99a-5p	None	
	hsa-mir-99a-3p	ALDH1A3, 7A1, LGR4, LRIG3	↑ $p< 0.05$
	[6]hsa-let-7c-5p	None	$p<0.01$
	hsa-let-7c-3p	ALCAM, ALDH7A1, 9A1, 8A1, 1B1, BMI1, LGR4, LGR5, LRIG1, LRIG3	NS

[redacted]			
Chr22	[6]hsa-let-7a-3-5p	None	p<0.01
	[7]hsa-let-7a-3-3p	None	p<0.01
	hsa-mir-4763-5p	ALDH5A1, 1L1, 7A1, 3B1, 3A2, 8A1, 1A2, 2, BMI1, LRIG3	NS
	[14]hsa-mir-4763-3p	ALDH3B2, 4A1, 5A1, 7A1, 3A2, LRIG3	NS
	hsa-let-7b-5p	None	p<0.01
	hsa-let-7b-3p	None	p<0.01
[redacted]			
**ChrX**	[3]hsa-mir-106a-5p broadly conserved	None	↑ p< 0.01
	hsa-mir-106a-3p	ALDH1L2, 1L1, 6A1, 7A1	NS
	hsa-mir-18b-5p broadly conserved	ALCAM	ND
	hsa-mir-18b-3p	ALDH6A1, LRIG2	ND
	[3]hsa-mir-20b-5p broadly conserved	None	NS
	hsa-mir-20b-3p	ALCAM, ALDH9A1, 1B1, 1A2, 3A2, 5A1, 1L2, 6A1, 7A1, LGR4, LRIG3	NS
	hsa-mir-19b-2-5p	ALDH1A2, 8A1, 18A1, 1L2, BMI1, LGR4	ND
	hsa-mir-19b-2-3p	None	ND
	hsa-mir-92a-2-5p	ALDH3B2, 5A1, 4A1, LGR4, LRIG2	ND
	[4]hsa-mir-92a-3p	None	ND
	[15]hsa-mir-363-5p	ALDH3A1, 4A1, 3B1, BMI1, LRIG2	ND
	[4]hsa-mir-363-3p	None	ND
[redacted]			
**ChrX**	hsa-let-7f-2-5p	ND	P<0.01
	[16]hsa-let-7f-2-3p	ALDH1A3, 3A2, 6A1, BMI1, LGR4, LRIG3	NS
	[6]hsa-mir-98-5p	None	↑ p< 0.01

	[7]hsa-mir-98-3p	ALDH6A1, LRIG2	NS

These miRNAs are also a member of the following broadly conserved families:

[1]miR-15-5p/16-5p/195-5p/424-5p/497-5p

[2]miR-16-2-3p/195-3p

[3]miR-17-5p/20-5p/93-5p/106-5p/519-3p

[4]miR-25-3p/32-5p/92-3p/363-3p/367-3p

[5]miR-96-5p/1271-5p

[6]let-7-5p/98-5p

[7]let-7a-3p/let-7b-3p/let-7f-1-3p/98-3p

[8]miR-99-5p/100-5p

[9]let-7a-2-3p/let-7g-3p

[10]miR-200c-5p/550a-3p

[11]miR-200bc-3p/429

[12]miR-141-3p/200a-3p

[13]miR-148-3p/152-3p

[14]miR-1207-5p/4763-3p

[15]mir-363-5p/6745

[16]let-7f-2-3p/1185-3p

NS= not significant, ND= no data, ↑ = increased expression in CRC, ↓ = decreased expression in CRC

Databases used: http://www.mirbase.org/ for identifying miRNA clusters, http://www.targetscan.org/vert_71/ for identifying isomiRs and miRNA targets, http://mirtv.ibms.sinica.edu.tw/ for clinical data on miRNA and miRNA targets
